# Implementing distancing in case of school reopening amid COVID-19 pandemic: Recommendations

**DOI:** 10.17179/excli2021-4142

**Published:** 2021-09-21

**Authors:** Mehrdad Askarian, Mohammad Hossein Taghrir, Alireza Estedlal, Taraneh Estedlal, Seyed Sajjad Tabei, Ardalan Askarian

**Affiliations:** 1 Department of Community Medicine, School of Medicine, Shiraz University of Medical Sciences, Shiraz, Iran; 2 Health Behavior Science Research Center, Shiraz University of Medical Sciences, Shiraz, Iran; 3 Student Research Committee, Shiraz University of Medical Sciences, Shiraz, Iran; 4 Department of Operative Dentistry, School of Dentistry, Tehran University of Medical Sciences, Tehran, Iran; 5 Medical Genetics Centre of Southern Iran, Shiraz University of Medical Sciences, Shiraz, Iran; 6 Student, College of Arts & Science, University of Saskatchewan, Saskatoon, Canada

## ⁯

***Dear Editor,***

Since December 2019, Coronavirus disease 2019 (COVID-19) began its journey worldwide, and compulsory changes were imposed on our daily life. Attempts to reduce the burden of this pandemic are mostly limitation measures, including imposing curfews, restricting public gatherings, limiting social interactions, imposing travel bans, and closing schools. 

The rationale for closing schools was based on past experiences from Influenza, which were reported to be effective (Litvinova et al., 2019[7]; Jackson et al., 2014[5]). Due to the remarkable dearth of related data and uncertainty regarding the transmissibility and morbidity factors of the Severe Acute Respiratory Syndrome-Coronavirus 2 (SARS-CoV-2), school closures as a preemptive action to protect school-aged children seemed to be compelling and was thought to be likely beneficial for impeding the virus transmission chain.

School closure was first implemented across China after the new year holidays (Tian et al., 2020[9]). On March 18, 2020, the United Nations Educational, Scientific and Cultural Organization (UNESCO) estimated that 107 countries had implemented a school closure strategy and predicted that roughly half of all students -approximately 862 million- will be affected by the COVID-19 (Viner et al., 2020[10]). Apart from the mental health problems that were imposed on children, school closure has led to the elimination of routine programs such as free nutrition and programs, school-based vaccination programs, school nursing, etc. Such programs are to promote equity amongst marginalized communities (Armitage and Nellums, 2020[1]).

Apart from the mentioned consequences, the effectiveness of such policy should be subjected to criticism. If we take a look at the Severe Acute Respiratory Syndrome (SARS) outbreak, the effectiveness of school closure policies is debatable. Most of the studies showed that it did not contribute to curbing the outbreak (Viner et al., 2020[10]). In the current pandemic, several studies, mostly from mainland China reported that measures ranging from quarantine to social distancing were effective, and school closure was part of these measures (Tian et al., 2020[9]; Lai et al., 2020[6]). However, in the aforementioned studies, the clear impact of school closure was not identified. By contrast, a population-based analysis from the United States showed that school closure had led to a significant decline in the incidences and the consequent mortality rates (Auger et al., 2020[2]). 

On the other hand, a noticeable number of studies revealed that the COVID-19 outbreak rate is lower amongst children. The role of transmission in schools is limited, and students do not act as the disease's reservoir (Munro and Faust, 2020[8]). Does this raise the question as to how long schools should stay closed?

Some disease transmissions within schools are inevitable. Hitherto, it was stated that the pathogenesis and the natural course of this disease are less profound amongst the pediatric population than adults and that the risk of severe illness is greatly diminished in the younger age groups unless they have other comorbidities and underlying disease (CDC, 2020[3]). Since schools play a major role in the education and nurturing of the students, their reopening with special considerations, including better hygiene practices and social distancing measures, appears to be logical. Therefore, going back to school with elevated levels of precaution and proactive initiatives to restrict a person-to-person transmission will be the new norm, at least for the time being. Executing distancing seems to be the most challenging measure, as it may necessitate additional funds and resources. Besides, vaccine approval for adolescents is another issue, and only a few developed countries like the US have recently given green light for children to be vaccinated (CDC, 2021[4]). Having said that, the continuation of precautionary measures such as social distancing seems to be the only tool at the disposal of policymakers and community health experts. 

To find the recommendations related to guidelines on distancing in the school setting for reopening in the COVID-19 pandemic era, we conducted a focused review of 22 roadmaps or frameworks of school reopening published by other countries or the US states on November 28, 2020 [Supplementary information]. To find the mentioned papers, we had to search *via* Google, since these are government documents and there is no related paper in the name of school reopening roadmap, guideline, or framework in the databases of PubMed, Scopus, and EMBASE. Two authors extracted the recommendations independently, and the senior author resolved the differences. Then, the extracted recommendations were summarized and categorized into six subgroups, as provided in Table 1(Tab. 1). Of note, this study was approved by the institutional board review and research ethics committee with the following numbers, respectively: 23595 and IR.SUMS.MED.REC.1399.331.

Finally, implementing distancing is of major importance in the process of school reopening to ensure students' health and safety as well as teachers and staff. We believe that our findings will shed light on the school reopening challenges, and the students can be protected if appropriately implemented.

## Conflict of interest

The authors declare no conflict of interest.

## Supplementary Material

Supplementary information

## Figures and Tables

**Table 1:**
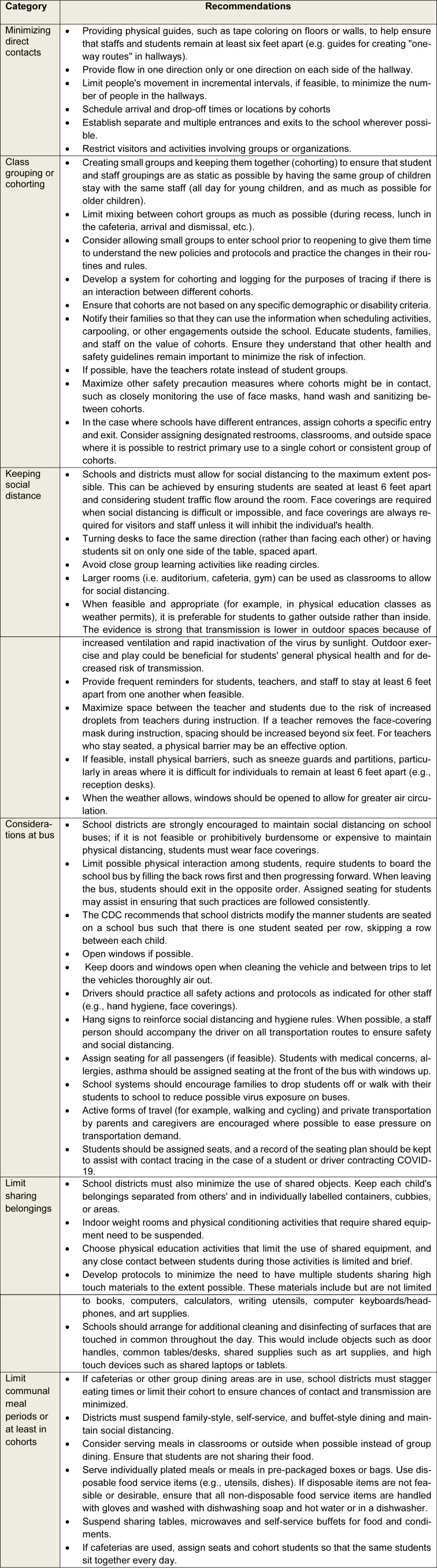
Recommendations on implementing distancing retrieved from school reopening roadmaps
