# Supplementary material for: Implementing distancing in case of school reopening amid COVID-19 pandemic: Recommendations
Source: EXCLI J. 2021 Sep 21;20:1407–11. doi: 10.17179/excli2021-4142 (PMC8564904; doi:10.17179/excli2021-4142)
Supplement: Supplementary information [file EXCLI-20-1407-s-001.pdf]

EXCLI Journal 2021;20:1407-1411 – ISSN 1611-2156
Received: August 01, 2021, accepted: September 01, 2021, published: September 21, 2021

Supplementary information to:

Letter to the editor:

IMPLEMENTING DISTANCING IN CASE OF SCHOOL REOPENING AMID COVID-19 PANDEMIC: RECOMMENDATIONS

Mehrdad Askarian MD, MPH[1,2], Mohammad Hossein Taghrir MD[1,*], Alireza Estedlal MD[3], Taraneh Estedlal DDS[4], Seyed Sajjad Tabei[5], Ardalan Askarian[6]

1 Department of Community Medicine, School of Medicine, Shiraz University of Medical Sciences, Shiraz, Iran
2 Health Behavior Science Research Center, Shiraz University of Medical Sciences, Shiraz, Iran
3 Student Research Committee, Shiraz University of Medical Sciences, Shiraz, Iran
4 Department of Operative Dentistry, School of Dentistry, Tehran University of Medical Sciences, Tehran, Iran
5 Medical Genetics Centre of Southern Iran, Shiraz University of Medical Sciences, Shiraz, Iran
6 Student, College of Arts & Science, University of Saskatchewan, Saskatoon, Canada

* **Corresponding author**: Mohammad Hossein Taghrir, MD. Department of Community Medicine, School of Medicine, Shiraz University of Medical Sciences, Shiraz, Iran; E-mail: mhtaghrir@gmail.com

http://dx.doi.org/10.17179/excli2021-4142

This is an Open Access article distributed under the terms of the Creative Commons Attribution License (http://creativecommons.org/licenses/by/4.0/).

The roadmaps and frameworks that were reviewed are available as following:

Access date to all is November 28, 2020

1- **Alabama**

https://www.tcss.net/cms/lib/AL01001644/Centricity/Domain/6779/Roadmap%20for%20Reopening%20Schools%20June%202020%20v14.pdf

2- **Arizona**

https://azdhs.gov/covid19/documents/schools/k-12-school-guidance-covid-19.pdf

3- **California Department of Education**

https://www.cde.ca.gov/ls/he/hn/documents/strongertogether.pdf

4- **California Department of Public Health**

https://files.covid19.ca.gov/pdf/guidance-schools--en.pdf

5- **Connecticut**

https://portal.ct.gov/-/media/SDE/COVID-19/CTReopeningSchools.pdf

6- **Delaware**

https://www.doe.k12.de.us/cms/lib/DE01922744/Centricity/Domain/600/guidance_fall2021.pdf

7- **Georgia**

https://www.georgiainsights.com/uploads/1/2/2/2/122221993/covid_guidancegeorgia_schools_08_12_2020njn.pdf

8- **Indiana**

https://www.doe.in.gov/sites/default/files/news/june-5-class-document.pdf?utm_content=&utm_medium=email&utm_name=&utm_source=govdelivery&utm_term=

EXCLI Journal 2021;20:1407-1411 – ISSN 1611-2156
Received: August 01, 2021, accepted: September 01, 2021, published: September 21, 2021

9- Kentucky

https://education.ky.gov/comm/Documents/Reopening%20Guid-ance%20%20051520kf_tkt%20421pm%20TM.pdf

10- Massachusetts

https://www.bostonherald.com/wp-content/uploads/2020/06/DESE-Initial-Fall-Reopening-Guidance-vFF.pdf

11- Michigan

https://www.michigan.gov/documents/whitmer/MI_Safe_Schools_Roadmap_FI-NAL_695392_7.pdf

12- Minnesota

https://educa-tion.mn.gov/mdeprod/idcplg?IdcService=GET_FILE&dDocName=MDE032934&RevisionSe-lectionMethod=latestReleased&Rendition=primary

13- Nevada

http://www.doe.nv.gov/uploadedFiles/ndedoenvgov/content/News__Media/Press_Re-leases/2020_Documents/NevadaDepartmentofEducationPathForwardPlanRe-sponsetoCOVID-19.pdf

14- New Jersey

https://www.nj.gov/education/reopening/NJDOETheRoadBack.pdf

15- North Carolina

https://docs.google.com/document/d/1z5Mp2XzO-OPkBYN4YvROz4YOyNIF2UoWq9EZfrjvN4x8/edit?ts=5ee01202#

16- Ohio

http://education.ohio.gov/getattachment/Topics/Reset-and-Restart/Reset-Restart-Guide.pdf.aspx?lang=en-US

17- Ontario

https://www.ontario.ca/page/guide-reopening-ontarios-schools

18- Pennsylvania

https://www.education.pa.gov/Schools/safeschools/emergencyplanning/COVID-19/SchoolReopeningGuidance/ReopeningPreKto12/Pages/default.aspx

19- South Carolina

https://scdhec.gov/sites/default/files/media/document/2021.2022-School-Guidance-Booklet-09.10.21.pdf

20- Texas

https://tea.texas.gov/sites/default/files/covid/covid19-SY-20-21-Public-Health-Guidance.pdf

21- UNESCO

https://en.unesco.org/news/framework-school-reopening

22- Washington

https://www.k12.wa.us/sites/default/files/public/workgroups/Reopening%20Washington%20Schools%202020%20Planning%20Guide.pdf
